# 气相色谱-负化学源质谱法测定船舶压载水中4种三卤甲烷

**DOI:** 10.3724/SP.J.1123.2022.01003

**Published:** 2022-06-08

**Authors:** Guoshen HU, Hong WANG, Keyao YU, Weijian SHEN, Yan HOU, Meiquan JI, Yiming ZHU, Wen TIAN, Xidong LI

**Affiliations:** 1.南京海关动植物与食品检测中心, 江苏 南京 210019; 1. Animal, Plant and Food Inspection Center, Nanjing Customs, Nanjing 210019, China; 2.江阴海关综合技术服务中心, 江苏 江阴 214440; 2. Jiangyin Customs Comprehensive Technical Service Center, Jiangyin 214440, China

**Keywords:** 顶空进样, 负化学源, 气相色谱-质谱法, 三卤甲烷, 船舶压载水, headspace injection, negative chemical ionization (NCI), gas chromatography-mass spectrometry (GC-MS), trihalomethanes (THMs), ship ballast water

## Abstract

我国每年的船舶压载水排放量巨大,压载水中含有浮游生物、病原体及其幼虫或孢子等,若处理不当,会对排放水域的生态环境造成严重影响。排放压载水前常使用电解法对其进行处理,电解产生的次氯酸钠溶液,能有效杀灭残余的微生物。但电解后会产生副产物三卤甲烷(THMs),其对人体有一定的健康风险,建立船舶压载水中三卤甲烷的测定方法具有重要意义。该研究建立了采用气相色谱-负化学源质谱(GC-NCI-MS)测定船舶压载水中4种三卤甲烷(包括三氯甲烷、二氯一溴甲烷、一氯二溴甲烷、三溴甲烷)的分析方法。船舶压载水样品经过顶空进样技术处理后,通过DB-5MS UI毛细管色谱柱(30 m×0.25 mm×1.0 μm)分离,气相色谱-负化学源质谱仪测定,在选择离子扫描(SIM)模式下分析,采用外标法进行定量。4种三卤甲烷在0.2~50 μg/L范围内线性关系良好,相关系数(*r*)≥0.995,定量限(*S/N*=10)为0.1~0.2 μg/L,在0.2、0.5、2.0 μg/L 3个加标水平下,4种THMs的平均回收率为90.3%~106.8%,相对标准偏差(RSD)为1.4%~6.2%。该方法准确、稳定、可靠,可用于测定船舶压载水中4种THMs的含量。使用建立的测定方法对36个船舶压载水进行测定,三溴甲烷、二溴一氯甲烷、一溴二氯甲烷与三氯甲烷的检出率分别为83.3%、69.4%、22.2%和19.4%,检出值分别为34.25~221.5 μg/L、3.52~41.87 μg/L、1.52~8.56 μg/L和0.02~5.46 μg/L。

## Abstract (en)

Ship ballast water can control the roll, trim, and draft of the ship, and thus ensuring the balance and stability of the ship in the course of sailing, and playing a vital role in the safe navigation of ships. The annual discharge of ship ballast water is very large in China. About three to five billion cubic meters of ship ballast water is discharged into offshore or inland waters every year. This water contains plankton, pathogens, and their larvae or spores. If not be handled appropriately, this will have a serious impact on the ecological environment of the discharge waters. Ballast water is usually treated by electrolysis before being discharged. Sodium hypochlorite can be generated, which can kill microorganisms; however, the by-products trihalomethanes (THMs) are cytotoxic and biotoxic. Studies have shown that THMs may cause fetal growth retardation, spontaneous abortion, or death. The concentration of THMs in drinking water is closely related to the risk of bladder cancer death. Hence, it is important to establish a method for the determination of THMs in ship ballast water. The four kinds of THMs are chloroform, dichlorobromomethane, chlorodibromomethane, and tribromomethane. At present, ship ballast water is mostly analyzed by gas chromatography (GC) using an electron capture detector (ECD) or by gas chromatography-mass spectrometry (GC-MS). Given the low boiling point of THMs, headspace injection and purge-and-trap can be used. Gas chromatography-negative chemical ionization-mass spectrometry (GC-NCI-MS), was adopted. NCI is a soft ionization technique that shows special response to compounds bearing electronegative elements or groups. THMs contain electronegative chlorine atoms and bromine atoms. Therefore, NCI is a good choice for their analysis. The samples were processed by the headspace injection technique. The NaCl content in 10 mL sample was optimized in headspace injection. The results showed that 3.0 g NaCl was the most suitable dosage. The analytes were separated on a DB-5MS UI capillary-column (30 m×0.25 mm×1.0 μm). The target compounds were quantified by using the external standard method in selected ion monitoring (SIM) mode. The four THMs were not only well separated but also showed a high response at 0.2 μg/L. The four THMs showed good linear relationships in the range of 0.2-50 μg/L, with correlation coefficients≥0.995. The limits of quantification (LOQs, *S/N*=10) were 0.1-0.2 μg/L, and the average recoveries of the four THMs were 90.3%-106.8% at the three spike levels of 0.2, 0.5, and 2.0 μg/L. The relative standard deviations were 1.4%-6.2%. The LOQs of the THMs in the GB/T 5750.8-2006 Standard Test Method of Drinking Water Organic Matter Index are 0.3-6.0 μg/L. It can be seen that the LOQs of the THMs are greatly reduced in this study. The proposed method is accurate, stable, and reliable, and it can be used for monitoring the four THMs in ship ballast water. The method was applied for the detection of 36 ship ballast water samples. In all cases, the detection rates of tribromomethane, chlorodibromomethane, dichlorobromomethane, and chloroform were 83.3%, 69.4%, 22.2%, and 19.4%, respectively. The detection values of tribromomethane, chlorodibromomethane, dichlorobromomethane, and chloroform were 34.25-221.5 μg/L, 3.52-41.87 μg/L, 1.52-8.56 μg/L, and 0.02-5.46 μg/L, respectively. Based on the analysis of several ship ballast water samples (electrolytic water), it was concluded that the greater the number of bromine atoms in the THMs, the higher are the detection rate and detection value in ship ballast water. Compared to chloroform, tribromomethane is more harmful to living beings. China has acceded to the International Convention on Ship Ballast Water and Sediment Control and Management. There is an urgent need to establish analysis methods with high sensitivity, good stability, and high accuracy in addition to determining standards and regulations for ship ballast water.

船舶压载水是指控制船舶横倾、纵倾和吃水的一种水,能够保证船舶在航行过程中的平衡与稳定,对船舶的安全航行有着至关重要的作用^[1,2]^。船舶通常需要在港口水域或者海水水域,将压载水压入或排出船舱。当船舶压入压载水后,水中含有的浮游动植物、微生物、有机污染物以及无机污染物也随之进入压载舱中^[2]^。当船舱内的压载水被排出时,这些有害物质也随之排放至异地海域,若处理不当,将对排放海域的生态环境造成严重影响^[3]^。

统计数据显示^[4]^,每年约有30~50亿立方米的船舶压载水被排放至近海或内陆水域,对排放地的生态影响不容小觑。国际组织高度重视压载水对生态环境的影响,2004年国际海事组织(IMO)通过了《国际船舶压载水和沉积物控制与管理公约》^[1]^,于2017年9月8日正式生效。我国在2018年10月22日正式加入《国际船舶压载水和沉积物控制与管理公约》,该公约于2019年1月22日起对我国正式生效。各航运国也纷纷开展了对船舶压载水处理和分析技术的研究。

电解法是一种处理压载水的方法之一,是将海水引入电解装置,电解产生高浓度的次氯酸钠溶液,其能够有效杀灭经过滤后残余的浮游生物、病原体及其幼虫或孢子等。目前电解法技术已经很成熟,在工业用水、生活用水以及废水处理上被广泛运用,但是电解可能产生副产物,例如三卤甲烷^[2]^。

三卤甲烷包括三氯甲烷(CHCl_3_)、二氯一溴甲烷(CHCl_2_Br)、一氯二溴甲烷(CHClBr_2_)和三溴甲烷(CHBr_3_)。压载水被电解后产生的三卤甲烷,其形成原理与饮用水消毒时三卤甲烷的生成原理类似,都是由氯及其化合物与水中的天然有机物反应而生成^[5]^。三卤甲烷具有细胞毒性和生物毒性,对人体有一定的健康风险。有研究表明,三卤甲烷会导致胎儿生长迟缓、自然流产和死亡等不良后果,同时饮用水中的三卤甲烷浓度与膀胱癌死亡风险有着密切关系^[6,7]^。因此世界各国都对饮用水中三卤甲烷的含量做了严格规定,我国颁布了GB 5749-2016《生活饮用水卫生标准》,规定了饮用水中三氯甲烷、二氯一溴甲烷、一氯二溴甲烷和三溴甲烷的限量分别为60、100、60、100 μg/L^[8]^。我国CJ/T 206-2005《城市供水水质标准》也规定了三卤甲烷总量的限量(100 μg/L)^[9]^。美国环保署(USEPA)也规定了饮用水中的三卤甲烷总和不得超过80 μg/L^[9]^。目前尚无船舶压载水中三卤甲烷的标准限量规定。

目前,水中三卤甲烷的测定多采用气相色谱法(配备电子俘获检测器(ECD))^[10,11]^和气相色谱-质谱法^[12,13]^,前处理方法多是根据三卤甲烷沸点较低的特点而采用顶空进样技术^[14,15]^和吹扫捕集法^[12,13]^。顶空进样技术操作简单,稳定性好,吹扫捕集较顶空技术相比,具有取样量小和富集效果好的特点,但仪器设备成本远高于顶空进样技术^[14,16]^。气相色谱仪(配备ECD)对具有电负性的化合物会产生信号,且化合物的电负性越强,响应就越大,适用于含有卤素元素的三卤甲烷^[17]^。但气相色谱仪的定性能力较差,对于压载水这类复杂的基质往往会因为基质干扰的问题造成假阳性。而质谱技术因其定性能力强的特点,已被广泛运用。根据已有文献^[12,13]^,水中的三卤甲烷在采用气相色谱-质谱法测定时,大多使用电子轰击(EI)离子化技术,但该技术容易受到复杂的基质成分以及色谱柱流失的干扰造成定量不准确。从三卤甲烷的分子结构来看,这类化合物均含有氯原子及溴原子,因而可以采用负化学源(NCI)作为电离方式。因为NCI作为一种软电离技术仅对具有电负性元素或基团的化合物有响应,且随着电负性的增加,其响应也显著增强,但对基质成分和柱流失几乎没有响应,具有较强的抗干扰能力^[18]^,适用于压载水中三卤甲烷的测定。本研究采用顶空进样与NCI-GC-MS在线联用检测技术,建立了测定压载水中4种三卤甲烷的分析测定方法。

## 1 实验部分

### 1.1 仪器、试剂与材料

6890N-5973C气相色谱-质谱仪(配有NCI),7697A顶空进样器,顶空进样瓶及带聚四氟乙烯衬垫螺口瓶盖,均购自美国Agilent Technologies公司。

4种三卤甲烷混合标准溶液(100 μg/mL,坛墨质检科技股份有限公司),甲醇(色谱纯,美国HoneyWell公司),氯化钠(分析纯,国药集团化学试剂有限公司)于使用前400 ℃烘烤4 h。

船舶电解压载水样品来源于江阴海关综合技术服务中心,取样自国内港口。样品取样完成后均在实验前密封避光,4 ℃冷藏储存。

### 1.2 标准溶液的配制

标准储备液:准确吸取1 mL 100 μg/mL的4种三卤甲烷混合标准溶液于10 mL棕色容量瓶中,用甲醇定容至刻度线,配制成质量浓度为10 μg/mL的混合标准储备液,4 ℃条件下避光保存,有效期为3个月。

标准中间溶液:准确移取上述4种三卤甲烷标准储备液1.0 mL于10 mL棕色容量瓶中,用甲醇定容至刻度线,配制成质量浓度为1 μg/mL的混合标准溶液,4 ℃条件下避光保存,有效期为3个月。

系列标准工作液:分别准确吸取适量混合标准中间液,用蒸馏水稀释配制成0.2、0.5、2、5、10、50 μg/L系列标准工作液,需现用现配。

### 1.3 样品采集及前处理

采样时,先加入0.3 g抗坏血酸于150 mL棕色采样瓶中,取水样至满瓶,加盖密封,4 ℃条件下避光保存,7 天内完成测定。

准确量取10 mL样品于顶空瓶中,并加入3 g氯化钠固体后,迅速盖上顶空瓶盖,待分析。若分析结果超出线性范围,则使用蒸馏水适当稀释后,再按照上述步骤进行前处理后,待仪器分析。

### 1.4 分析条件

#### 1.4.1 气相色谱

色谱柱:Agilent DB-5MS UI石英毛细管色谱柱(30 m×0.25 mm×1.0 μm);进样口温度:230 ℃;柱流速:1.0 mL/min;进样方式:脉冲分流进样,分流比25∶1;载气:高纯氦气(纯度≥99.999%);程序升温条件:35 ℃保持3 min, 30 ℃/min升至125 ℃保持1 min, 40 ℃/min升至230 ℃保持5 min;传输线温度:250 ℃。

#### 1.4.2 顶空进样

平衡温度:60 ℃;定量环温度:70 ℃;传输线温度:80 ℃;加热时间:30 min;进样量:800 μL。

#### 1.4.3 质谱

离子源:负化学源;离子源温度:150 ℃;四极杆温度:150 ℃;反应气:甲烷(>99.9%),流量2 mL/min;离子化电压由自动调谐后得到;采集方式:选择离子监测(SIM)模式,具体参数见表1。

**表1:** 4种三卤甲烷的保留时间、定量离子和定性离子

Compound	Retention time/min	Quantitative ion (*m/z*)	Qualitative ion (*m/z*)
CHCl_3_	2.282	35.0	37.0
CHCl_2_Br	3.715	78.9	80.9
CHClBr_2_	5.461	78.9	80.9
CHBr_3_	6.552	78.9	80.9

## 2 结果与讨论

### 2.1 仪器方法的选择

目前未见采用GC-NCI-MS测定三卤甲烷的相关文献。根据三卤甲烷的分子结构,采用负化学源测定时,其抗干扰能力和灵敏度会优于气相色谱-质谱法(EI源)和气相色谱法(配备ECD)^[18]^。本研究采用1.4.2节顶空进样参数处理压载水加标样品,采用GC-NCI-MS技术进行分析,得到的总离子流色谱图见图1。4种三卤甲烷不仅得到了较好的分离,而且在0.2 μg/L水平下依旧能够得到较高的响应。

**图1:**
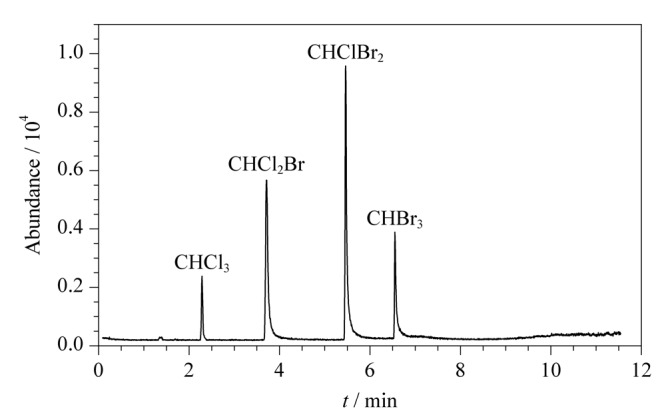
船舶压载水加标样品(0.2 μg/L)中4种三卤甲烷的总离子流色谱图

### 2.2 前处理条件的优化

#### 2.2.1 氯化钠的加入量

在压载水样品中加入适量的氯化钠,能够减少水相中三卤甲烷的溶解度,提高气相中三卤甲烷的浓度,从而提高分析的灵敏度。本研究分别在10 mL压载水加标样品中加入0.5、1.0、2.0、3.0、4.0、5.0 g的氯化钠,GC-NCI-MS分析后记录每个样品的峰面积,氯化钠加入量和峰面积关系见图2。

**图2:**
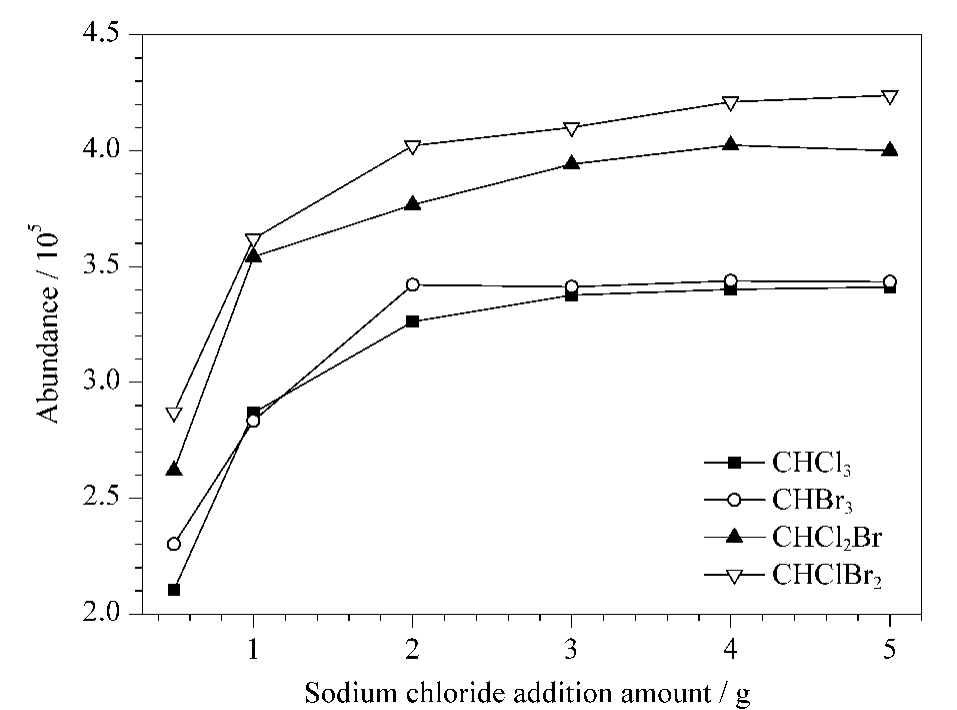
4种三卤甲烷峰面积与氯化钠加入量的关系

可以发现,当氯化钠加入量由0.5 g不断增加时,4种三卤甲烷的峰面积起初均明显增加,当氯化钠的加入量为3.0 g时,样品中的氯化钠处于饱和状态,此时4种三卤甲烷的峰面积趋于稳定,继续增加氯化钠的加入量后,4种三卤甲烷的峰面积不再明显增加,故选取3.0 g的加入量最为合适。

#### 2.2.2 保存方式对三卤甲烷的影响

所有船舶压载水样品均在采样时加入了适量的抗坏血酸,并置于棕色玻璃瓶中,冷藏避光保存。本研究考察了船舶压载水中三卤甲烷含量与放置天数的关系,分别于第1、2、3、4、5、6、7天测定了船舶压载水中三卤甲烷含量,实验结果见表2。可以看出,在前4天三卤甲烷的测定值较为稳定,第5天后三卤甲烷的测定值发生较明显的变化,但变化不大,变化的差值与第一天数值相比小于10%。由此可以看出,船舶压载水中三卤甲烷在7天内检测是可以的,在前4天检测测定值最为稳定。

**表2:** 4种三卤甲烷含量与放置天数的关系

Compound	Contents/(μg/L)						
	1 d	2 d	3 d	4 d	5 d	6 d	7 d
CHCl_3_	3.54	3.58	3.55	3.50	3.47	3.30	3.20
CHCl_2_Br	6.58	6.53	6.62	6.55	6.22	6.01	5.97
CHClBr_2_	15.72	15.50	15.62	15.22	15.33	14.60	14.33
CHBr_3_	167.42	168.65	163.22	155.97	154.33	153.98	150.89

### 2.3 方法学验证

#### 2.3.1 标准曲线、检出限和定量限

采用外标法定量,以定量离子的峰面积为纵坐标(*y*),工作溶液的质量浓度为横坐标(*x*),建立线性回归方程,回归方程与相关系数见表3,结果显示,*r*为0.9956~0.9998,表明4种THMs在0.2~50 μg/L范围内线性关系良好。以信噪比(*S/N*)=3确定检出限,*S/N*=10确定定量限,得到仪器的检出限为0.02~0.06 μg/L,定量限为0.1~0.2 μg/L,见表3。GB/T 5750.8-2006《生活饮用水标准检验方法有机物指标》中三卤甲烷的定量限为0.3~6.0 μg/L,由此看出本研究大大降低了三卤甲烷的定量限,有着更好的灵敏度。

**表3:** 4种三卤甲烷的回归方程、相关系数、检出限和定量限

Compound	Regression equation	*r*	LOD/ (μg/L)	LOQ/ (μg/L)
CHCl_3_	*y*=0.461073*x*-0.029321	0.9956	0.06	0.2
CHCl_2_Br	*y*=0.343835*x*-0.019627	0.9963	0.02	0.1
CHClBr_2_	*y*=0.226119*x*-0.005216	0.9993	0.02	0.1
CHBr_3_	*y*=0.145196*x*-0.044452	0.9998	0.06	0.2

*y*: peak area of quantitative ion; *x*: mass concentration of the compound, μg/L.

#### 2.3.2 加标回收率与精密度

选取船舶压载水阴性样品进行加标回收试验。分别添加混合标准溶液,添加水平为0.2、0.5、2.0 μg/L,每个添加水平做6个平行样品。按照1.3节前处理条件处理后,采用GC-NCI-MS技术测定。计算平均回收率与相对标准偏差(RSD)。结果显示,船舶压载水中4种THMs的平均回收率为90.3%~106.8%,RSD为1.4%~6.2%,见表4。

**表4:** 船舶压载水中4种三卤甲烷在3个水平下的加标回收率和相对标准偏差(*n*=6)

Compound	0.2 μg/L			0.5 μg/L			2.0 μg/L		
	Recovery/%	RSD/%		Recovery/%	RSD/%		Recovery/%		RSD/%
CHCl_3_	98.8	4.2		101.4	2.3		90.3		2.6
CHCl_2_Br	103.0	5.3		105.1	1.7		93.4		2.1
CHClBr_2_	101.7	6.2		104.6	2.0		94.8		1.7
CHBr_3_	103.1	5.0		106.8	1.4		100.2		2.3

### 2.4 实际样品分析

本研究按照已建立的分析方法,对36个压载水样品(电解水)进行测定,结果显示三溴甲烷的检出率最高,为83.3%,测定值为34.25~221.5 μg/L,其次是二溴一氯甲烷,检出率为69.4%,测定值为3.52~41.87 μg/L,一溴二氯甲烷与三氯甲烷的检出率相对较低,分别为22.2%和19.4%,测定值为1.52~8.56 μg/L和0.02~5.46 μg/L。从结果来看,三溴甲烷的检出率和检出值大于二溴一氯甲烷、一溴二氯甲烷、三氯甲烷。目前尚未有针对海水电解后三卤甲烷生成机理的深入研究。根据已有文献可以发现^[19⇓-21]^,影响三卤甲烷生成的因素包括三卤甲烷前体的类型和浓度、氯的含量、温度、pH、溴化物浓度、反应时间以及光照条件等。同时,不同的研究者都根据自己的实验结果,建立了三卤甲烷生成机理的一元或多元的数学模型。这些数学模型反应机理并不完全一致,但都能解释一定的实验现象。若要解释电解后的海水中三卤甲烷的生成机理,还需要进一步研究。

## 3 结论

本研究采用顶空进样技术进行前处理,利用气相色谱-负化学源质谱技术测定船舶压载水(电解水)中4种三卤甲烷的含量,同时也能够满足饮用水中三卤甲烷的测定要求。因负化学源仅对压载水中含有电负性较强的氯原子或溴原子的三卤甲烷有专属响应,气相色谱-负化学源质谱法体现出了高选择性和高灵敏度。

通过分析多个船舶压载水样品(电解水),发现了三卤甲烷中溴原子越多的化合物,在船舶压载水中检出率和检出值就越大。相较于三氯甲烷,三溴甲烷的毒性更高,对生物的危害性更大。我国在加入《国际船舶压载水和沉积物控制与管理公约》后,尽早建立灵敏度高、稳定性好、准确度高的分析方法,并制定出相应的标准法规,才能走在各航运国的前列,也能够为国内压载水处理商提供技术支持。
